# Redox‐Active Halide Catholytes for Solid‐State Lithium Batteries

**DOI:** 10.1002/advs.202514215

**Published:** 2025-11-09

**Authors:** Guang Sun, Zhenyou Song, Yiming Dai, Zuke Xiao, Xueying Zheng, Wei Luo

**Affiliations:** ^1^ Institute of New Energy for Vehicles School of Materials Science and Engineering Tongji University Shanghai 201804 China

**Keywords:** extra capacity, halide, redox‐active catholyte, solid‐state battery, solid‐state electrolyte

## Abstract

All‐solid‐state Li batteries that incorporate halide solid‐state electrolytes (SEs) promise the safety, thermal stability, and high energy density needed for electric vehicles and grid‐scale renewable energy storage. However, a substantial fraction of these electrochemical inert SEs is used as catholytes within the composite cathodes to sustain continuous Li‐ion pathways, adding “dead weight” that does not contribute to the overall capacity. This review highlights the emerging redox‐active halide catholytes, specifically Li containing transition metal halide that combine reversible redox chemistry with efficient mixed ionic–electronic conductivity. When integrated into composite cathodes, these materials contribute to an additional 20–50% reversible capacity, and simultaneously lowers the electronic transport tortuosity. Drawing on insights from solid‑state ionics and electronic band structure, the design principles for achieving mixed ionic–electronic conductivity are first outlined, and then summarize recent progress on Fe‐, V‐, and Ti‐based redox‐active halide catholytes, along with their dynamic behavior during cycling. Finally, the conclusion outlined further directions for redox‐active halide catholytes, including the discovery of new materials, anion‑sublattice engineering, and the elucidation of dynamic interface evolution, as well as the exploration of anionic‑redox processes.

## Graphical abstract

This review highlights emerging redox‐active halide catholytes that merge reversible transition‐metal redox chemistry with mixed ionic–electronic conductivity. Integrating these materials into composite cathodes of all‐solid‐state batteries boosts reversible capacity by 20–50% and reduces electronic transport tortuosity. Design principles, recent advances, and future directions in material discovery are comprehensively discussed.

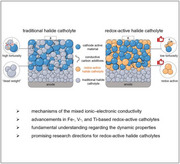

## 1 Introduction

The goal of achieving a carbon‐neutral society has intensified efforts to develop sustainable and renewable power technologies. While advances in lithium‐ion batteries have yielded higher energy density (e.g., 350–500 Wh kg^−1^), safety concerns emerge that originate from the use of flammable liquid electrolytes and organic polymeric separators.^[^ ^1^ ^]^ To mitigate these hazards, researchers are exploring strategies such as aqueous electrolytes, non‐flammable electrolytes, and solid‐state electrolytes (SEs). Among these, all‐solid‐state Li batteries (ASSLBs) based on inorganic SEs have recently been deemed a promising solution, as they substantially reduce the risks of flammability and electrolyte leakage by replacing volatile liquid components, although metallic lithium and oxygen‐based cathodes can still pose potential thermal hazards.^[^ ^2^, ^3^ ^]^ In this context, the realization of exciting benefits of ASSLBs greatly depends on the physicochemical properties of SEs. Ever since the breakthrough work by Asano et al. on Li_3_YCl_6_ and Li_3_YBr_6_,^[^ ^4^ ^]^ emerging halide SEs have attracted considerable attention owing to their high Li‐ion conductivities (≈10^−2^ S cm^−1^) at room temperature (RT), comparable to that of conventional liquid electrolytes.^[^ ^5^, ^6^ ^]^ Furthermore, the excellent oxidative stability (up to 4 V vs Li^+^/Li) and compressibility (e.g., >90% density under 250−350 MPa) make them particularly suitable as catholytes, functioning as Li‐ion conductive additives within the composite cathodes.^[^ ^7^, ^8^, ^9^, ^10^ ^]^

**Figure 1:**
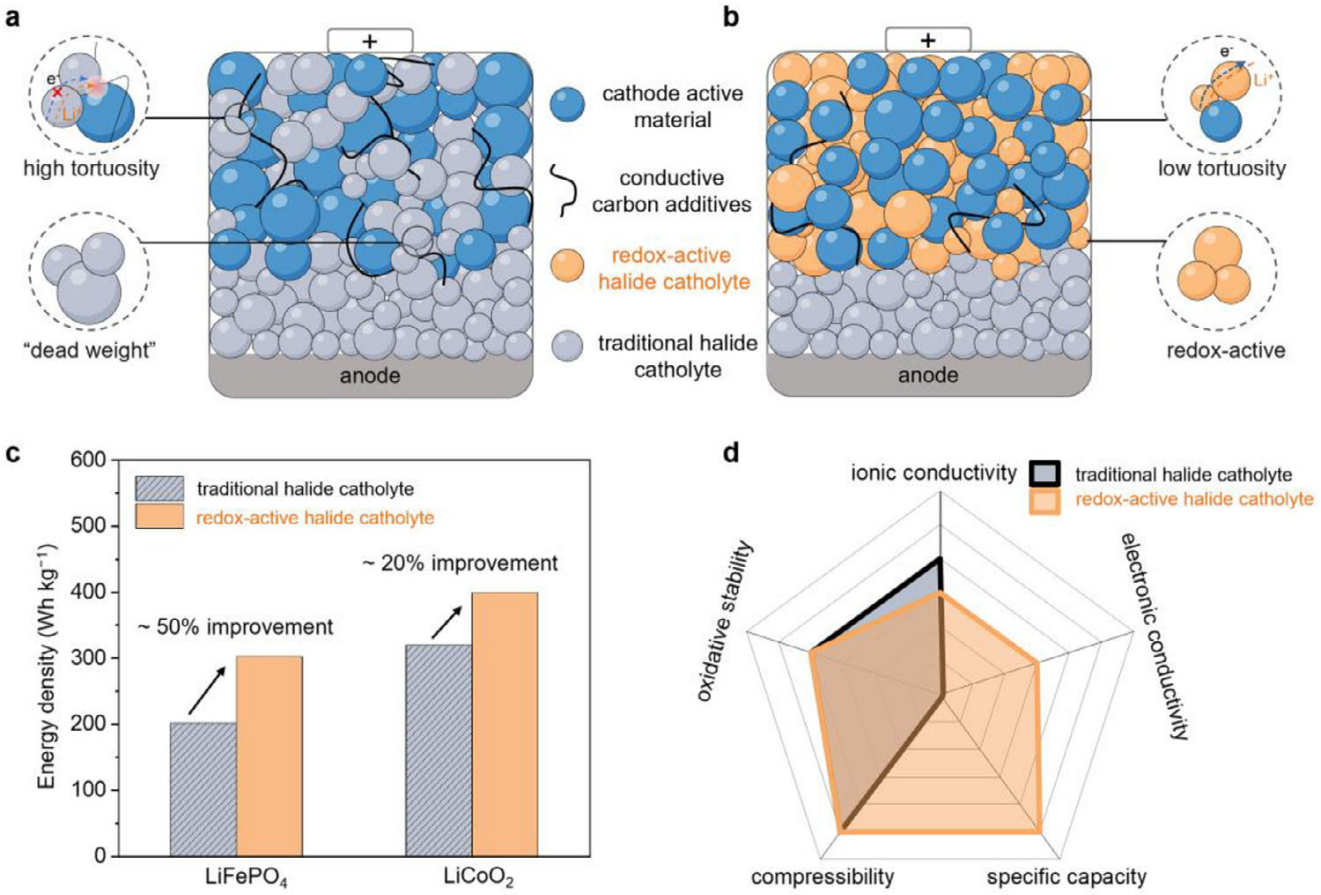
Functionality and potential advantages of redox‐active halide catholytes. Structural schematic of ASSLBs composed of a) traditional halide catholytes and b) redox‐active halide catholytes. c) Comparison of energy density regarding the LiFePO_4_ (ref. [12]) and LiCoO_2_ (ref. [13]) composite cathodes, incorporate with either traditional halide catholytes or redox‐active halide catholytes. d) Spider plots comparing the key performances of traditional halide catholytes with redox‐active halide catholytes.

However, traditional halide catholytes are electrochemically inactive, as they incorporate metal cations in a fixed oxidation state (e.g., Y^3+^, In^3+)^ that do not participate in redox reactions. Consequently, materials such as Li_3_YCl_6_ and Li_3_InCl_6_ are employed solely to facilitate ion transport, while not contributing to any electrochemical capacity (**Figure** **1**a). Thus, these substantial fractions of “dead weight”, typically 15–50 wt.% of composite cathodes, significantly reduce both the gravimetric and the volumetric energy densities.^[^ ^11^ ^]^ To overcome these limitations (Figure 1b), recent studies have pivoted to explore redox‐active halide catholytes in which the inert metal cation is incorporated or substituted with redox‐active transition metals (e.g., Fe, V, Ti). Once coupled with commercial cathodes such as LiFePO_4_ and LiCoO_2_, these redox‐active catholytes can deliver extra reversible capacity, enabling potential improvements in energy density of ≈20–50% (Figure 1c).^[^ ^12^, ^13^ ^]^ Furthermore, as shown in Figure 1d, redox‐active halide catholytes retain high ionic conductivity, oxidative stability, and compressibility, while their intrinsic electronic conductivity (10^−5^–10^−7^ S cm^−1^) is orders of magnitude higher than that of the traditional halide catholytes (10^−9^–10^−10^ S cm^−1^), potentially reducing the tortuosity of electron transport pathways, and thereby accelerating the redox reaction kinetics. Consequently, the integration of redox‐active halide catholytes represents a paradigm shift in the design of next‐generation ASSLBs.

Herein, we review recent progress in the emerging field of redox‐active halide catholytes, emphasizing their potential on increasing battery energy density, as well as reducing the tortuosity of the electron transport pathways in ASSLBs. We begin by outlining the fundamental mechanisms of the mixed ionic–electronic conductivity from the perspectives of solid‐state ionics and electronic band structure, respectively. Following this, we highlight recent advancements in Fe‐, V‐, and Ti‐based redox‐active halide catholytes, together with a fundamental understanding regarding the dynamic properties during cycling. Looking forward, we call for attention designing new materials, exploring dynamic properties and anionic redox chemistry during cycling, and the precise tuning of the anion sublattice, all of which are crucial for further development of redox‐active halide catholytes.

## 2 Ionic and Electronic Transport Properties

Developing suitable halide catholytes with inherent mixed ionic–electronic conductivity represents a revolutionary direction for the next‐generation ASSLBs, offering the prospect for lowering Li^+^/e^−^ transport tortuosity and enhancing the redox reaction kinetics.^[^ ^14^, ^15^ ^]^ In conventional composite cathodes, achieving simultaneous percolation for Li‐ion and electron transport is challenging because these pathways are provided separately by the catholyte and conductive carbon. In such systems, multiple solid–solid interfaces between the active material, catholyte, and carbon can exacerbate interfacial side reactions and lead to rapid impedance growth during cycling.^[^ ^16^, ^17^, ^18^ ^]^ In contrast, mixed‐conducting halide catholytes reduce the reliance on conductive carbon by providing both transport pathways within one phase, thereby decreasing interfacial complexity.^[^ ^19^, ^20^, ^21^ ^]^ Furthermore, the seamless integration of ionic and electronic pathways also simplifies cathode fabrication, and potentially allows for the use of thick cathodes, which effectively increases the battery areal capacity and energy density. Here, we would elucidate the fundamental origins of the mixed ionic–electronic conductivity in redox‐active halide catholytes from the perspectives of solid‐state ionics and energy band theory, respectively.

### 2.1 Li‐Ion Conductivity in Redox‐Active Halide Catholytes

Efficient ion transport network within composite cathodes poses a prerequisite for the rate capabilities of ASSLBs, which hinges greatly on the intrinsic Li‐ion conductivity of redox‐active catholytes.^[^ ^22^, ^23^, ^24^ ^]^ Yet, there is now a substantial gap regarding Li‐ion conductivity as compared to that of the traditional halide catholytes, despite of their similar crystal structures and conduction pathways. To overcome such limitation, research still leans heavily on intuition‐driven trials and errors, yet several design principles have been established with the discovery of sulfide and oxide counterparts, involving the tailored highly conductive SEs crystal structures to enable reversible redox activity.^[^ ^25^, ^26^ ^]^

**Figure 2:**
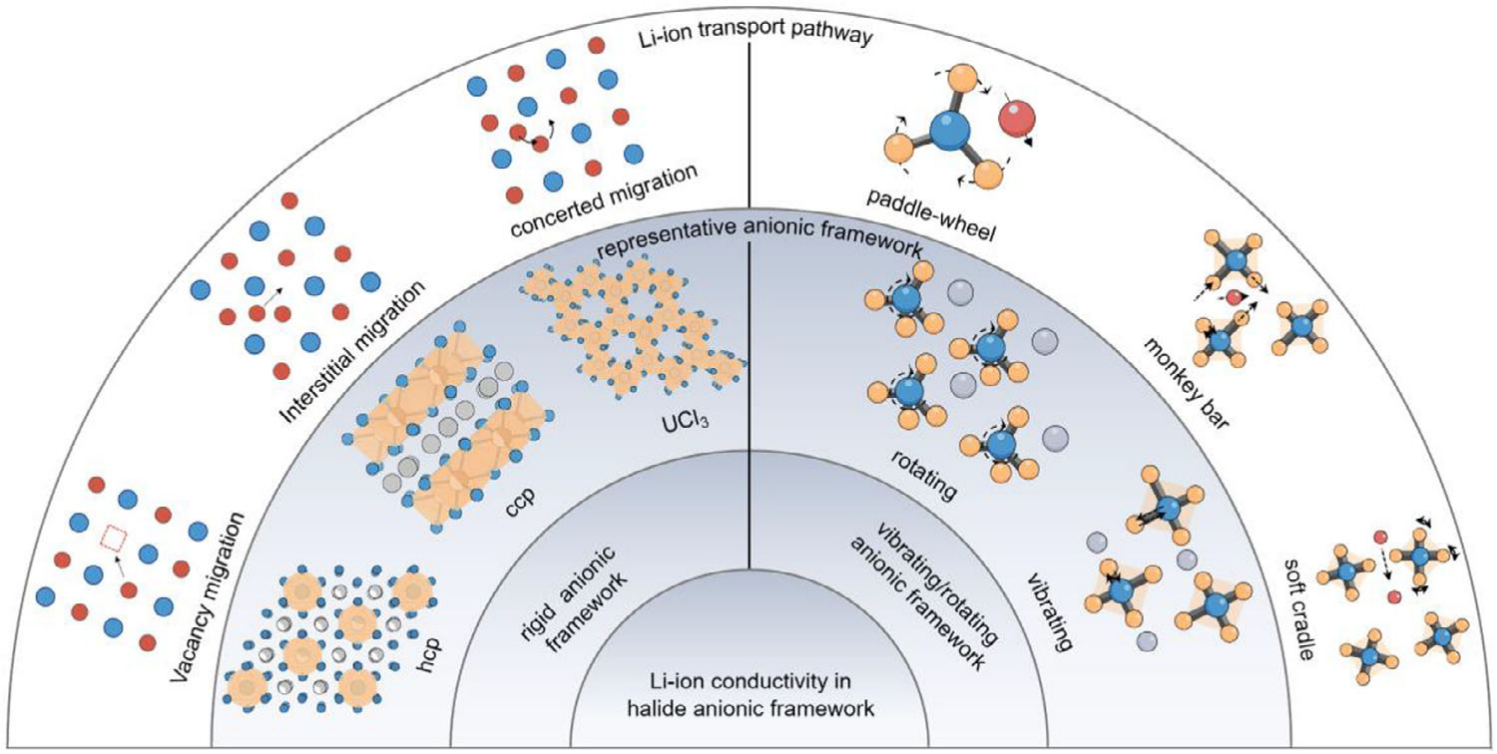
Schematic overview of the Li‐ion transport pathway in the representative halide anionic framework, highlighting the effect of rigid or vibrating/rotating halide anionic frameworks on Li‐ion transport.

In this section, we dive into the interaction between the representative crystal structures and the Li‐ion transport pathway (**Figure** **2**). From the perspective of solid‐state ionics, the anion framework provides accessible site for Li‐ion hopping, where fundamental relationship between anion packing and vacancy‐dependent diffusion pathways are desired to aid the design of novel halide catholytes with high ionic conductivity. Furthermore, anion motion is also critical in recently reported amorphous halide solid electrolytes, where fast Li‐ion conduction occurs via a liquid‐like mechanism enabled by local anion vibrating/rotating. Here, we present their respective effects on Li‐ion transport depending on whether the anionic frameworks are either relatively rigid or vibrating/rotating.

#### 2.1.1 Li‐Ion Transport in Rigid Anionic Frameworks

With respect to the rigid anion frameworks, the topology of the anion sublattices manifests the key factor in determining bottleneck size, Li‐ion diffusion pathway, and the activation energy.^[^ ^27^, ^28^, ^29^ ^]^ Notably, the cutting‐edge sulfide SEs usually adopt a body‐centered cubic (bcc) anion sublattice, providing both large interstitial sites and a bottleneck, which allows for a lowest activation barrier and a highest ionic conductivity.^[^ ^30^, ^31^ ^]^ However, Cl^−^ are generally less polarizable than S^2−^, they priorly adopt closely packed sublattices such as hexagonal close‐packed (hcp) and cubic close‐packed (ccp) anion sublattice.^[^ ^32^ ^]^ For instance, in Li_3_VCl_6_ and Li_3_TiCl_6_, a ccp anion lattice has been confirmed by X‐ray diffraction and the subsequent Rietveld refinement.^[^ ^12^, ^33^ ^]^ Note that the central transition metal takes a six‐coordinated octahedron with the anion in either the ccp or hcp anionic sublattice. Besides, as the central‐cation radius grows, its coordination can shift from the usual sixfold to ninefold. For instance, the UCl_3_‐type structure consisting of edge‐sharing tricapped trigonal prism of UCl_9_ that provides 1D channels with large bottleneck size is beneficial for rapid Li‐ion conduction.^[^ ^34^, ^35^ ^]^ Based on the representative anionic framework, two fundamental migration mechanisms are found through numerous ab initio computational investigations: 1) vacancy diffusion, in which neighboring Li‐ion migrates into a vacant site; 2) interstitial migration between interstitial sites.^[^ ^36^, ^37^ ^]^

While the state‐of‐the‐art halide SEs have a Li‐ion conductivity of 12 mS cm^−1^ experimentally,^[^ ^5^ ^]^ molecular dynamics simulations show that halide SEs based on the chloride anionic sublattices have the potential to achieve higher lithium ionic conductivity (14–29 mS cm^−1^),^[^ ^38^ ^]^ suggesting that there are still undiscovered theoretical mechanisms for further optimization. Given that concerted Li‐ion migration has been demonstrated in several halide SEs,^[^ ^39^ ^]^ it is intuitive to consider this mechanism as a key contributor to ionic transport in halide catholytes. In this context, Mo's group proposed a multi‐ion concerted migration mechanism based on a rigid anionic framework,^[^ ^40^ ^]^ rather than on isolated ion hopping as is typical in solid. Using ab initio modeling, they discovered that concerted migration of multiple ions has a lower energy barrier because of the stronger ion‐ion interactions between ions located at the high‐energy and low‐energy sites. From this point of view, the ions located at the high‐energy sites migrate downhill, which cancels out a part of the energy barrier felt by other uphill‐climbing ions. Interestingly, this provides an additional mechanistic aspect on the aliovalent substitution, that is, non‐Li cations with lower valences occupy the low‐energy sites, forcing the mobile Li ions to occupy the high‐energy sites.^[^ ^41^ ^]^ Therefore, designing advanced halide catholytes requires balancing anion topology, vacancy concentration, and the site occupancy of mobile Li‐ions.

#### 2.1.2 Li‐Ion Transport Based on Anionic Motion

In contrast to the rigid anionic framework, which confines Li‐ion to narrow, high‐energy barrier diffusion channels, Li‐ion transport based on anionic motion features a non‐close‐packed anionic framework that emphasizes the impact of the cation‐anion interaction. However, anionic motion often comes at the expense of amorphizing the crystal structure, which poses a great challenge for probing the long‐range transport of Li‐ion.^[^ ^42^, ^43^, ^44^ ^]^ Advanced techniques, such as molecular dynamics simulations, atom pair distribution functions, and X‐ray absorption spectroscopy can offer a wealth of valuable information about the local structure, and thus reveal the influence of anion vibrational/rotational dynamics on the Li‐ion transport through mechanisms like monkey bar,^[^ ^45^ ^]^ paddle‐wheel,^[^ ^46^ ^]^ and soft cradle effect.^[^ ^47^ ^]^ With respect to the vibrating anionic framework in glassy LiTaCl_6_, the large‐amplitude vibrations of Cl^−^ anion within TaCl_6_ unit was related to fast Li‐ion hopping, enabled by the facile breaking and reforming of Li‐Cl bonds, named with monkey bar mechanism. As the name implies, the flexible anionic framework effectively acts as a “monkey bar”, allowing Li ions to swing from one site to another with significantly reduced migration barriers.

Compared to the prevalence of vibration phenomena of anionic sublattice, rotation of anionic framework was once thought to be almost impossible at RT, since great energy barrier must be surmounted to overcome the strong static electricity. In fact, the idea of anion rotation effect has been proposed for more than 40 years, when several inorganic crystalline materials with polyanions, such as SO_4_2^−^ and PO_4_3^−^ adopt “plastic” phases at high temperature (over 1000 °C) that exhibit high ion conductivity.^[^ ^48^, ^49^ ^]^ While the superionic conductivity of these high‐temperature “plastic” phases was ascribed to the rapid reorientation of polyanions, known as the “paddle‐wheel mechanism”, neither experimental nor theoretical proof was found at that time. More recent studies have applied the concept of anion rotational flexibility to halide systems and demonstrated that the motion of M–octahedral chains enhances cation diffusion. In this context, Ceder's group identified a “soft cradle” effect that relates Li hopping behavior with the tilting of polyhedral in the LiMXCl_4_ family,^[^ ^47^ ^]^ implying that the local rotational freedom of anions can directly contribute to a long‐range ionic transport. Such anionic motion opens promising avenues for the rational design of halide catholytes, particularly in amorphous or glassy phases that inherently support higher vibrational and rotational freedom than their crystalline counterparts. Recent studies have highlighted that certain amorphous chloride SEs exhibit comparable or even superior ionic conductivities to crystalline phases, largely attributed to their flexible and dynamically disordered anion sublattices.^[^ ^43^, ^50^, ^51^, ^52^ ^]^ For instance, aliovalent substitution shows powerful amorphous formation ability to preferentially act as bridging atoms to provide a fulcrum for anion rotation.^[^ ^53^, ^54^ ^]^ Thus, these findings highlight the potential of designing advanced halide catholytes that intentionally combine controlled structural disorder with a flexible anionic framework to promote high ionic conductivity.

### 2.2 Electronic Conductivity in Redox‐Active Halide Catholytes

**Figure 3:**
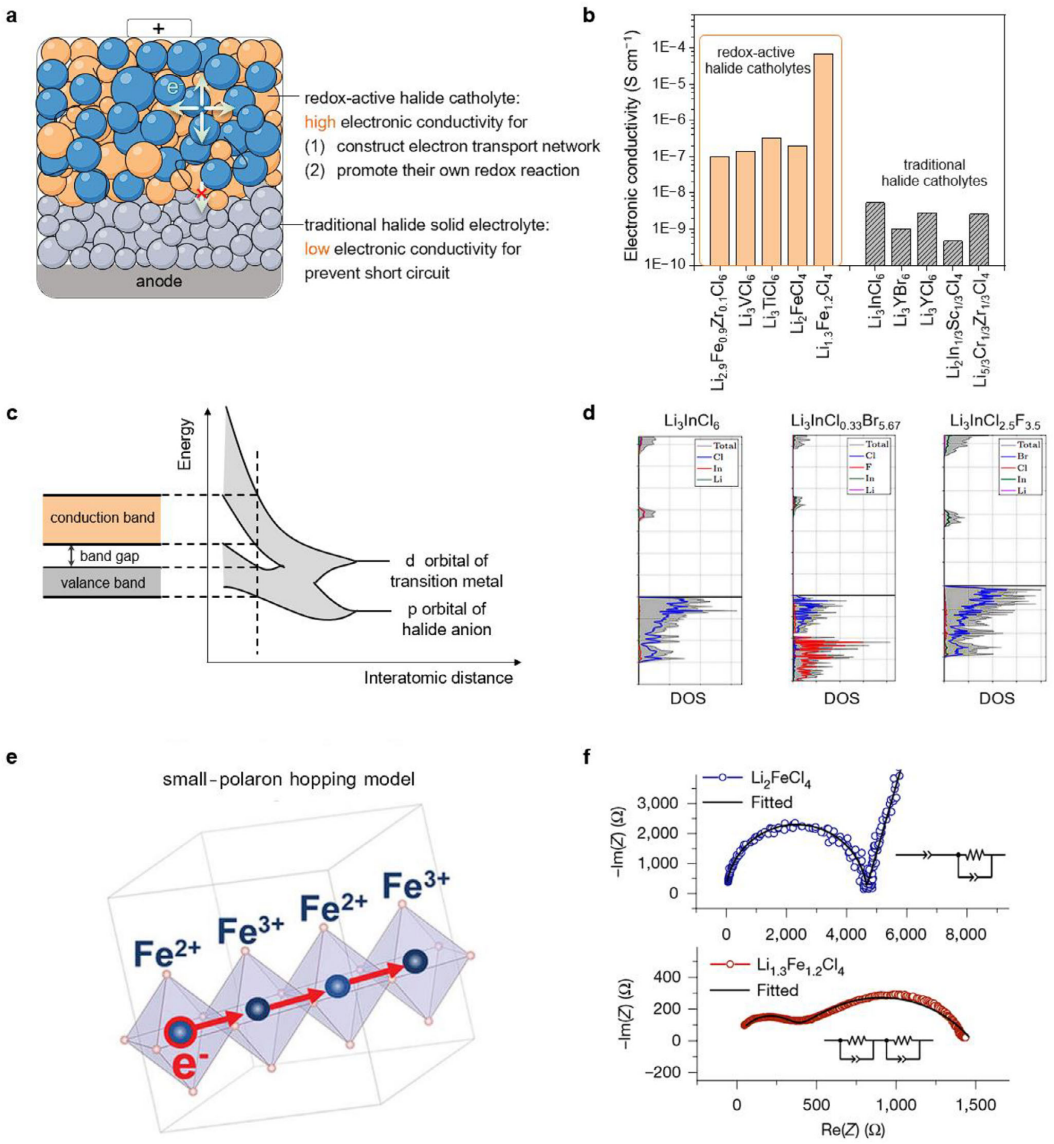
a) Schematic illustration of functionality and b) a bar chart comparing electronic conductivity between redox‐active halide catholytes and traditional halide solid electrolytes. c) Schematic band formation based on transition metal and anion coordination. d) Density of states of Li_3_InCl_6_, Li_3_InCl_0.33_Br_5.67_, and Li_3_InCl_2.5_F_3.5_. Reproduced with permission.^[61]^ Copyright 2024, IOP Publising. e) Schematic illustration of Fe^2+^/Fe^3+^ cations leads to nearest neighbor small‐polaron hopping model. Reproduced with permission.^[65]^ Copyright 2020, Wiley‐VCH GmbH. f) Electrochemical impedance spectroscopy (EIS) showing the impedance (Z) of Li_2_FeCl_4_ (top) and Li_1.3_Fe_1.2_Cl_4_ (bottom) in Nyquist plots, along with their fitted equivalent circuits (insets). Reproduced with permission.^[66]^ Copyright 2025, Springer Nature.

SEs and catholyte, as different functional components in ASSLBs (**Figure** **3**a), have diametrically opposed requirements on electronic conductivity. The ideal SEs should have minimal electronic conductivity to avoid gradual self‐discharge, Li‐dendrite growth and short circuits failure.^[^ ^55^, ^56^, ^57^ ^]^ In contrast, high intrinsic electronic conductivity is an advantage for redox‐active halide catholytes, as it facilitates the formation of efficient electron transport network and promotes their own redox reactions at the three‐phase interface.^[^ ^58^, ^59^ ^]^ Figure 3b compares the intrinsic electronic conductivity of the redox‐active halide catholytes with that of the conventional halide catholytes, revealing values that are two to five orders of magnitude higher. However, this electronic conductivity is still significantly lower than that of the electron‐conductive additives commonly used today (10^5^ S cm^−1^). In this review, we discuss the electronic band structure as a powerful framework to understand and design redox‐active halide catholyte with high electronic conductivity.

Generally, electronic conductivity is an intrinsic property of materials that is governed by electronic‐structure descriptors such as the band gap and the density/mobility of carriers. In halide catholytes, the valence and conduction bands arise primarily from overlap between transition‐metal d orbitals and anion p orbitals (Figure 3c).^[^ ^60^, ^61^ ^]^ Thus, introducing transition‐metal cations with partially filled d shells can generate states within the band gap, narrowing the gap and increasing the carrier concentration. Additionally, these trends are captured by the partial density of states (pDOS): a larger pDOS at the Fermi level indicates greater availability of electronic carriers and correlates with higher electronic conductivity.

From the perspective of the halide anion, p‐orbital energy increases monotonically down the halogen group. This upward shift elevates the valence‐band maximum of the hybrid orbitals formed with transition‐metal d states, narrows the band gap, and consequently enhances the electronic conductivity. For instance, the band structure of inorganic halide perovskites with the general formula ABX_3_ (A = inorganic monovalent cation, B = divalent cation, X = Br^−^, or I^−^) can be tuned by varying the halide composition in mixed‐anion ABBr_3−x_I_x_ compounds, which are known as highly efficient light‐harvesting materials for solar cells.^[^ ^62^, ^63^ ^]^ Likewise, in chalcogenide‐based transition metal materials, MCh_2_ (ch = O, S, Se, Te), σ_e_ continuously increases from O to Te due to the increased anionic band top.^[^ ^64^ ^]^ Guided by this principle, halogen substitution in Li_3_InCl_6_ provides a direct handle to tailor the electronic structure. Figure 3d compares the density of states (DOS) for Li_3_InCl_6_, Li_3_InCl_0.33_Br_5.67_, and Li_3_InCl_2.5_F_3.5_. Compared with F substitution, Br incorporation elevates the valence‐band maximum, increases the DOS near the Fermi level, and narrows the band gap, which is consistent with a potential enhancement in electronic conductivity.^[^ ^61^ ^]^

Besides, the design of redox‐active halide catholytes with high electronic conductivity crucially depends on the transition metal elements such as Ti^3+^ (d^1^) and V^3+^ (d^2^), which creates narrow, partially filled conduction bands and introduces defect levels within the bandgap. Conversely, closed‑shell cations such as Zn^2+^ (d^10^) are generally unsuitable, since their fully‐filled d manifold lies well below the Fermi level, and therefore supplies no partially filled states to sustain electronic transport. Furthermore, mixed‑valence transition metals such as Fe^3+^/Fe^2+^ promote small‑polaron hopping via a thermally activated local electron‑transfer mechanism (Figure 3e).^[^ ^65^ ^]^ Because the hopping occurs between neighboring iron sites, it remains operative even in materials with relatively wide band gaps. As shown in Figure 3f, the electronic conductivity of Li_1.3_Fe_1.2_Cl_4_ was determined to be 6.98 × 10^−5^ S cm^−1^ using the EIS method, which is three orders of magnitude higher than that of Li_2_FeCl_4_.^[^ ^66^ ^]^ The higher electronic conductivity of Li_1.3_Fe_1.2_Cl_4_ relative to Li_2_FeCl_4_ can be rationalized by the combined effects of 1) an increased density and connectivity of Fe sites, which enhances small‐polaron concentration and percolation, and 2) a larger fraction of Fe^3+^/Fe^2+^ pairs that facilitates thermally activated local electron transfer, effectively lowering the apparent hopping barrier and thus increasing σ_e_. Consequently, tailoring energy band structure through careful coordination of n transition metal and anions is an effective strategy for designing redox‑active catholytes with intrinsically high electronic conductivity.

## 3 Reversible Redox Chemistries for Halide Catholytes

**Figure 4:**
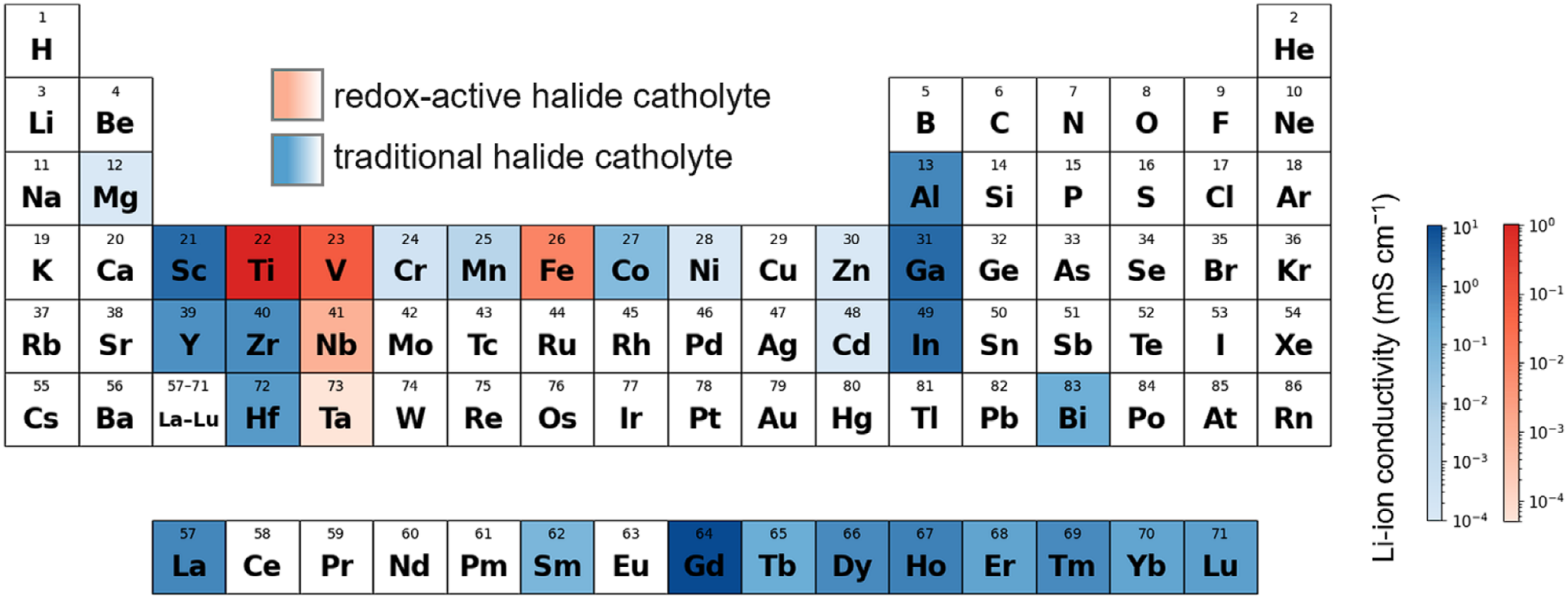
Periodic palette for the design of redox‐active halide catholytes. Elements are categorized by function: blue denote conventional halide catholytes, and yellow denote redox‐active halide catholytes. Within each category, darker shades indicate higher room‐temperature Li‐ion conductivity associated with compounds centered on the given cation. The Li‐ion conductivity and representative references used to construct the map are provided in **Table** **1**.

Since the advent of Li‐ion batteries, reversible transition‐metal redox chemistries have underpinned benchmark cathode materials such as LiCoO_2_, LiFePO_4_, and LiMn_2_O_4_.^[^ ^67^ ^]^ In these materials, transition‐metal cations gain or lose electrons, thereby providing capacity during electrochemical cycling. Similarly, a paradigm shift in halide catholytes design involves incorporating electrochemically active elements to obtain high specific capacity and electrochemical potential. However, selecting a suitable transition metal is non‑trivial because redox activity must be balanced against Li‑ion conductivity. **Figure** **4** illustrates this trade‑off: blue marks denote the conventional halide electrolytes, yellow marks the redox‐active catholytes; Within each color, darker shades correspond to higher Li‐ion conductivity for the given central cation. With respect to traditional halide catholytes, electrochemically inert elements, such as Al,^[^ ^68^ ^]^ In,^[^ ^69^ ^]^ and Sc^[^ ^70^ ^]^ are commonly chosen in order to achieve a high Li‐ion conductivity above 1 mS cm^−1^ as listed in Table 1. In contrast, catholytes incorporating redox‑active 3d metals such as Mn, Co, and Ni often exhibit lower Li‐ion conductivity because the tighter, more covalent M–Cl frameworks restrict Li⁺ diffusion.^[^ ^71^ ^]^ Although this narrows the pool of viable metals, the remaining ones still allow numerous compositional permutations. Among these, sustainable 3d transition metals such as Fe (Li_2.9_Fe_0.9_Zr_0.1_Cl_6_, Li_1.3_FeCl_4_), V (Li_3_VCl_6_), and Ti (Li_3_TiCl_6_) have received great attention as candidates to replace the more expensive In‐ or Y‐based halide catholytes. These examples provide a viable path toward high‑capacity, redox‑active halide catholytes, while retaining high Li‐ion conductivity of 10^−4^–10^−3^ mS cm^−1^, as discussed below.

**Table 1:** Room‐temperature Li‐ion conductivity of representative halide catholytes. For some compounds, (e.g., Li_2_CdCl_4_, Li_2_MgCl_4_, Li_2_ZnCl_4_, Li_6_CoCl_8_, Li_2_CrCl_4_, and Li_6_NiCl_8_), the Li‐ion conductivity was extrapolated from high‐temperature data.

Central cation	Chemical formula	Li‐ion conductivity [mS cm^−2^]	Refs.
Nb	LiNbCl_6_	1 × 10^−3^	[72]
Ta	LiTaCl_6_	6 × 10^−5^	[72]
V	Li_3_VCl_6_	7.5 × 10^−2^	[12]
Fe	Li_2_FeCl_4_	1 × 10^−2^	[73]
Ti	Li_3_TiCl_6_	1.04	[33]
Tm	Li_3_TmCl_6_	0.89	[74]
Yb	Li_3_YbCl_6_	0.32	[75]
Zr	Li_2_ZrCl_6_	0.81	[9]
Lu	Li_3_LuCl_6_	0.4	[76]
In	Li_3_InCl_6_	2.02	[7]
Sc	Li_3_ScCl_6_	3	[77]
Al	AlOCl‐LiCl	1	[42]
Sm	SmCl_3_‐0.5LiCl	0.12	[78]
Mn	Li_2_MnCl_4_	4 × 10^−3^	[79]
Bi	LiBiBr_4_	0.19	[80]
Cd	Li_2_CdCl_4_	1 × 10^−4^	[79]
Mg	Li_2_MgCl_4_	1 × 10^−4^	[79]
Zn	Li_2_ZnCl_4_	1 × 10^−5^	[81]
Co	Li_6_CoCl_8_	1 × 10^−3^	[82]
Cr	Li_2_CrCl_4_	1 × 10^−3^	[83]
Ga	2LiCl‐GaF_3_	3.2	[84]
Ni	Li_6_NiCl_8_	1 × 10^−4^	[85]
Hf	Li_2_HfCl_6_	0.5	[86]
La	Li_3_LaI_6_	1.25	[87]
Gd	Li_3_GdCl_3_Br_3_	11	[88]
Y	Li_3_YCl_6_	0.7	[4]
Er	Li_3_ErCl_6_	0.33	[89]
Tb	Li_3_TbCl_6_	0.22	[74]
Dy	Li_3_DyCl_6_	0.9	[74]
Ho	Li_3_HoCl_6_	1.3	[74]

### 3.1 Fe‐Based Redox‐Active Halide Catholytes

Fe is an attractive redox‐active element due to its high electrochemical potential, low toxicity, and abundance in the Earth's crust. Representative Fe‐based cathodes including LiFePO_4_, LiFeSO_4_F, and FeF_3_ exhibit desirable reversibility. Among halide counterparts, the low‐cost FeCl_3_ stans out as promising cathode for ASSLBs, delivering 558 Wh kg^−1^ at a materials cost only ≈2% of that of LiFePO_4_.^[^ ^90^ ^]^

Moreover, transition‐metal halides maintain Li⁺ diffusion coefficients (10^−10^ cm^−2^ s^−1^) throughout lithiation/delithiation process, indicating that their lithiated phases could function as efficient catholytes.^[^ ^91^, ^92^ ^]^ In fact, Li‐containing Fe‐based halides have a long history of research. Spinel Li_2_FeCl_4_ was first synthesized in the 1980s and showed a low ionic conductivity of ≈ 1 × 10^−5^ S cm^−1^ at 30 °C.^[^ ^93^ ^]^ In 2020, Tanibata et al. reported a cubic phase Li_2_FeCl_4_ with an improved ionic conductivity (2.1 × 10^−5^  S cm^−1^).^[^ ^94^ ^]^ When blended with 10 wt.% of electron‐conductive carbon additive, Li_2_FeCl_4_ underwent a one‐electron reaction at 3.6 V vs Li^+^/Li. However, its long‐term cycling stability has not been reported. Most recently, Li_2_FeCl_4_ was revisited as a cost‐effective and durable cathode, exhibiting a main lithiation plateau at ≈3.7 V vs Li^+^/Li with a specific capacity of 130 mAh g^−1^.^[^ ^73^ ^]^

**Figure 5:**
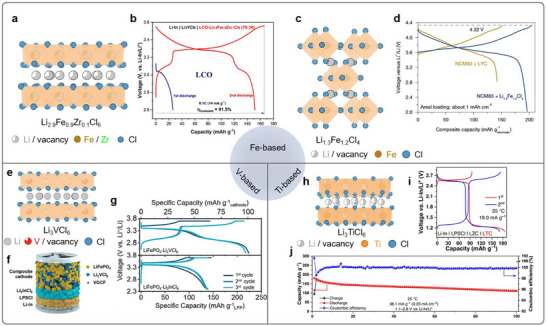
Reversible redox chemistries for Fe‐, V‐, and Ti‐based halide catholytes. a) Crystal structures of Li_2.9_Fe_0.9_Zr_0.1_Cl_6_. b) Voltage profiles of the first discharge and following charge and discharge under 0.1 C of Li–In|Li_3_YCl_6_|LCO‐Li_2.9_Fe_0.9_Zr_0.1_Cl_6_ (70:30) all‐solid‐state cells. Reproduced with permission.^[13]^ Copyright 2024, American Chemical Society. c) Crystal structures of Li_1.3_FeCl_4_. d) Crystal structure of Li_1.3_Fe_1.2_Cl_4_ with a *Cmmm* space group. d) The charge/discharge curves of the composite of NCM83/LYC and NCM83/Li_1.3_Fe_1.2_Cl_4_ plotted vs the capacity of the cathode composite at 25 °C and 0.05 C. Reproduced with permission.^[66]^ Copyright 2025, Springer Nature. e) Crystal structures of Li_3_VCl_6_. f) Schematic diagram of the all‐solid‐state cell configuration with LiFePO_4_‐Li_3_VCl_6_ composite cathode. Reproduced with permission.^[12]^ Copyright 2024, Wiley‐VCH GmbH. g) Discharge/charge curves of the LiFePO_4_‐Li_3_VCl_6_ and LiFePO_4_‐Li_3_InCl_6_ composite cathodes at 0.1 C. Reproduced with permission.^[12]^ Copyright 2024, Wiley‐VCH GmbH. h) Crystal structures of Li_3_TiCl_6_. i) The charge/discharge curves of the first two cycles and j) long‐term cycling performance of Li_3_TiCl_6_ composite cathode. Reproduced with permission.^[33]^ Copyright 2023, Springer Nature.

Bearing in mind the representative crystal structure of halide superionic conductors, Zhang et al. reported that Li_3_FeCl_6_ crystallises in a monoclinic phase analogous to that of the Li_3_InCl_6_ compounds (**Figure** **5**a).^[^ ^13^ ^]^ While mechanochemical synthesis of Li_3_FeCl_6_ yields a moderate Li‐ion conductivity (0.13 mS cm^−1^), partial substitution with Zr^4+^ expands the lattice framework and promotes Fe^3+^ occupancy at octahedral sites, thereby stabilizing the *C2/m* phase and improving the Li‐ion conductivity to 0.25 mS cm^−1^. Notably, a reversible capacity of 81 mAh g^−1^ was observed based on the Fe^2+^/Fe^3+^ redox couple. Consequently, a LiCoO_2_‐Li_2.9_Fe_0.9_Zr_0.1_Cl_6_ composite cathode delivers a high specific capacity of 153 mAh g^−^,^1^ providing ≈20% extra increment in energy density (Figure 5b). However, the electronic conductive additive is still required, due to the low electronic conductivity (2.16 × 10^−7^ S·cm^−1^ at 25 °C) of Li_2.9_Fe_0.9_Zr_0.1_Cl_6_. As mentioned earlier, coexisting of Fe^2+^ and Fe^3+^ can enhance the electronic conductivity via a small‐polaron hopping mechanism. Accordingly, Sun's group developed Li_1.3_Fe_1.2_Cl_4_ material with a space group of *Cmmm* (Figure 5c).^[^ ^21^ ^]^ By leveraging reversible Fe^2+^/Fe^3+^ redox, as well as the mixed Li^+^/e^−^ conductivities of 2.28 × 10^−4^ S cm^−1^ and 6.98 × 10^−5^ S cm^−1^ at 25 °C, respectively, the specific discharge capacity of the cathode composite increases from 141.9 (based on the total weight of NCM83 and Li_3_YCl_6_) to 196.1 mAh g^−1^ (NCM83 and Li_1.3_Fe_1.2_Cl_4_) at 0.05 C, achieving an electrode energy density of 725.6 Wh kg^−1^ (Figure 5d). Furthermore, the combination of fast Li^+^/e^−^ tansport and structural reversibility of Li_1.3_Fe_1.2_Cl_4_ further enable the additive‐free, all‐in‐one cathodes. Taken together, the sustainability and cost‐effectiveness of Fe‐based halide catholytes enhances their potential as practical and competitive catholytes, or even as homogeneous cathodes, for next‐generation ASSLBs.

### 3.2 V‐Based Redox‐Active Halide Catholytes

Vanadium has long received attention as a redox‐active transition metal for electrode materials because its multivalent chemistry readily spans oxidation states  from +3 to +5. This versatility enables the transfer of multiple electrons per vanadium center, potentially leading to higher specific capacity. Early studies in the 1980s have already explored the layered vanadium oxides such as V_2_O_5_, which was found to accommodate Li^+^ ions via intercalation with a high theoretical capacity (≈294 mAh g^−1^). However, their structural instability and capacity fading have limited the practical applications. To improve voltage and stability, researchers in the 2000s shifted their focus to polyanion vanadium compounds, leveraging the electron‐withdrawing inductive effect of highly electronegative tetrahedral anionic groups (e.g., PO_4_^3–^) to raise the operating voltage and stabilize high oxidation states. In parallel, vanadium‐based NASICON‐type phosphate (e.g., Na_3_V_2_(PO_4_)_3_) became attractive for sodium‐ion batteries, highlighting vanadium's broad applicability.^[^ ^95^ ^]^ Notably, vanadium redox activity in halide hosts was difficult to verify until dissolution was mitigated by super‐concentrated electrolytes and halide SEs.^[^ ^96^ ^]^ Inspired by this, our group proposed a redox‐active halide catholyte, Li_3_VCl_6_,^[^ ^12^ ^]^ which crystallizes in a monoclinic *C*2/*m* and exhibits a Li‐ion conductivity of 0.075 mS cm^−1^ at RT (Figure 5e). Moreover, Li_3_VCl_6_ delivers a reversible capacity of 80 mAh g^−1^ at ≈3 V vs Li⁺/Li based on the V^3+^/V^2+^ redox couple. When coupled with a traditional LiFePO_4_ cathode, the redox‐active Li_3_VCl_6_ catholyte enables an impressive capacity of 217.1 mAh g^−1^ based on the mass of LiFePO_4_, achieving ≈50% increase in the battery energy density over inactive catholytes (Figure 5f,g).

### 3.3 Ti‐Based Redox‐Active Halide Catholytes

From a historical view, initial studies in the 1990s highlighted the excellent thermodynamic stability of titanium, and its low reduction potential, making it particularly suitable for electrode materials. One of the earliest and most successful examples was the investigation of TiS_2_ layered sulfides, which enabled fast Li⁺ diffusion due to their 2D frameworks.^[^ ^97^ ^]^ Likewise, fast Li^+^ diffusion has been shown in LiTi_2_(PS_4_)_3_ (LTPS),^[^ ^98^ ^]^ which was first reported in 2008 as a cathode active material by Goodenough et al. With Ge/Se co‐doping, Li_1.75_Ti_2_(Ge_0.25_P_0.75_S_3.8_Se_0.2_)_3_ (LTG_0.25_SSe_0.2_) possesses considerable Li^+^/e^–^ conductivity of 0.22/242 mS cm^−1^, together with a specific capacity of 250 mAh g^−1^, manifesting a paradigm shift material to construct homogeneous electrode.^[^ ^60^ ^]^ With respect to halide catholytes, Wang et al. proposed the Li_3_TiCl_6_ with a *C2/m* space group as active materials, which shows a Li‐ion conductivity of 1.04 mS cm^−1^ at RT (Figure 5h).^[^ ^33^ ^]^ A composite cathode containing 95 wt.% of Li_3_TiCl_6_, and an additional of 5 wt.% carbon has delivered a capacity of 185 mAh g^−1^, with voltage plateaus corresponding to the Ti^3+^/Ti^4+^ and Ti^2+^/Ti^3+^ redox reactions at 2.53 and 1.24 V vs Li^+^/Li‐In, respectively (Figure 5i). The cell has retained a satisfactory capacity over extended cycling (Figure 5j). Although Li_3_TiCl_6_ was not used as a catholyte owing to its relative low electrochemical potential, its favorable crystal structure for Li‐ion transport makes it a promising candidate for further optimization through transition metal doping.^[^ ^99^ ^]^ Overall, these insights underscore the potential of titanium‐based halide in the design of advanced redox‐active halide catholytes.

### 3.4 Other Redox‐Active Halide Catholytes

The above‐mentioned findings point to a broader elemental space that can be exploited to simultaneously enhance Li‐ion conductivity and redox activity, especially those multivalent transition metal such as Nb and Ta.^[^ ^72^, ^100^ ^]^ In this context, Ridley et al. proposed an aliovalent substitutions concept, synthesizing a series of Na_2−x_M_x_Zr_1−x_Cl_6_ (0< x <1, M = Nb or Ta) solid‐solutions with Na_2_ZrCl_6_ (x = 0) and NaMCl_6_ (x = 1, M = Nb or Ta) as end‐member structures.^[^ ^100^ ^]^ As a result, this approach not only enhances the ionic conductivity compared to the respective end‐member compounds, but also triggers charge storage capacity by incorporating redox‐active Nb^5+^ and Ta^5+^. Therefore, partial replacement of electrochemically inert cations with redox‑active Nb or Ta emerges as a practical route to the redox‑active halide catholytes, marrying fast ion conduction with intrinsic electrochemical capacity, and offering a tunable balance between the two.

## 4 Dynamic Properties During Cycling

Fast charge transfer across the cathode active materials|catholyte interface and efficient mixed ionic–electronic conductivity within the bulk phase are the two essential prerequisite for redox‐active halide catholytes. However, in practical batteries, repetitive (de)lithiation processes induce phase evolution and microstructure reconfiguration that inevitably affect the interfacial contact and conductivity of the redox‐active catholytes. Therefore, a thorough characterization of their dynamic properties during cycling, including phase transformations, changes of mixed ionic‐electronic conductivity, and interfacial evolution, is critical for optimizing their performance. Here, we review current insights into these phenomena, and highlight key experimental techniques to interpret the dynamic properties of redox‐active halide catholytes.

### 4.1 Crystal Structural Evolution During (De)Lithiation

**Figure 6:**
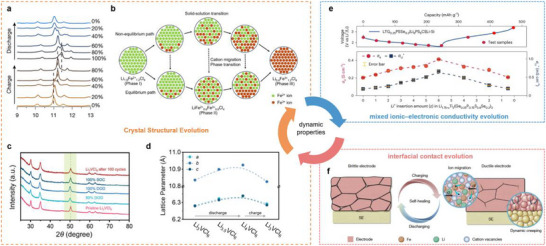
Dynamic properties of redox‐active halide catholytes during cycling. a) Ex situ XRD profile with an X‐ray wavelength of 0.3497 Å of Li_1.3_Fe_1.2_Cl_4_ at different states of charge and discharge. Reproduced with permission.^[66]^ Copyright 2025, Springer Nature. b) Schematic depiction of reaction paths in delithiation of Li_1.3_Fe_1.2_Cl_4_. Reproduced with permission.^[66]^ Copyright 2025, Springer Nature. c) XRD patterns of Li_3_VCl_6_ at different SOCs and DODs, and d) the corresponding changes of lattice parameters (a–c). Reproduced with permission.^[12]^ Copyright 2024, Wiley‐VCH GmbH. e) σ_Li+_ and σ_e_ of LTG_0.25_PSSe_0.2_ as a function of the electrochemically inserted Li^+^ content in the first cycle. Reproduced with permission.^[60]^ Copyright 2024, Springer Nature. f) Schematic of the brittle‐to‐ductile transition of the Li_x_Fe_1.2_Cl_4_ and the self‐healing behavior of the mechanical failures. Reproduced with permission.^[66]^ Copyright 2025, Springer Nature.

Phase transformations are ubiquitous in redox‐active materials, because Li‐ion insertion or removal imposes lattice strain that can drive solid‐solution, two‐phase, or even conversion reactions. Redox‑active halide catholytes incorporate multivalent transition metals to unlock extra capacity, and therefore experience pronounced local‑ and long‑range structural transformation during cycling. As revealed by ex situ XRD patterns at various SOCs (**Figure** **6**a), Li_1.3_Fe_1.2_Cl_4_ proceeds through a combination of solid‐solution and two‐phase mechanisms during lithiation: 1) initial oxidation of Fe^2+^ to Fe^3+^ causes average lattice parameter shrinkage, resulting in XRD peak shift to higher angles; 2) further Li^+^ extraction triggers sequential two‑phase transformations.^[^ ^21^ ^]^ Furthermore, the relative contribution of these pathways is rate‑dependent (Figure 6b), reflecting the competition between equilibrium and non‐equilibrium routes. In contrast, our group demonstrated that Li_3_VCl_6_ undergoes a purely solid–solution mechanism: its main diffraction peaks located at 2𝜃 = 30°, 35°, and 50° shift gradually to lower angles, as evidenced by ex situ XRD patterns (Figure 6c), with lattice parameters increasing monotonically during discharging (Figure 6d).^[^ ^12^ ^]^ While Li_3_VCl_6_ can accommodate one mole of Li per formula unit without detectable phase separation, other systems may exhibit irreversible phase transformations at deeper states of discharge. For instance, Li_3_TiCl_6_ undergoes delithiation through the growth of a Li‐deficient phase.^[^ ^33^ ^]^ Yet, further structure determination is quite challenging, as it requires a sample that is free from the original Li_3_TiCl_6_ phase. Therefore, advanced operando characterization techniques such as synchrotron X‐ray diffraction (SXRD) are crucial for monitoring real‐time phase evolution as the cell is cycled. SXRD can reveal subtle shifts in lattice parameters and the appearance of new phases, thus providing valuable insights into the stability and reversibility of redox‐active halide catholytes. Overall, clarifying phase evolution is essential for designing catholytes that combine high reversible capacity while sustaining Li‐ion pathways during long‐term cycling.

### 4.2 Mixed Ionic–Electronic Conductivity Evolution

To construct a continuous ionic–electronic transport network within the composite cathodes, redox‐active halide catholytes must deliver high mixed ionic–electronic conductivity in the pristine state, and sustain, or even enhance this conductivity during reversible redox cycling.^[^ ^15^, ^101^ ^]^ In principle, the insertion of additional Li⁺ ions modifies the carrier concentration and the distribution of Li vacancies, thereby directly affecting the ionic conductivity. On the other hand, the lithiation process of redox‐active halide catholytes, accompanied by continuous addition of electrons, is expected to narrow the bandgap and thereby increase the σ_e_. Thus, the delicate balance between ionic and electronic conductivity must be reassessed as the degree of lithiation evolves. As shown in Figure 6e, σ_Li+_ increases from 0.22 to 0.66 mS cm^−1^ as the number of inserted Li^+^ ions increases from 1 to 6, owing to the higher carrier concentration. Similarly, the σ_e_ monotonically rises from 242 to 412 mS cm^−1^ with progressive lithiation. To interpret these trends, it is useful to consider LiCoO_2_: pristine LiCoO_2_ is semiconducting, and delithiation progressively increases its metallic character as electrons are removed from the filled t_2_g band, leading to hole formation and higher electronic conductivity.^[^ ^102^ ^]^ Similarly, electronic states gradually appear near the Fermi level, and the bandgap of LTG_0.25_PS narrows from 0.749 to 0 eV during lithiation, suggesting a transition in conductive behavior from semiconducting to metallic. The interplay between ionic and electronic transport is further complicated by microstructural factors such as grain boundaries, phase interfaces, and lattice defect, which can act as either conduits or barriers depending on the local stoichiometry and charge state.^[^ ^103^, ^104^ ^]^ Advanced impedance spectroscopy and in situ conductivity measurements, including DC polarization and Hebb‐Wagner methods, are valuable tools for decoupling ionic and electronic contributions during battery operation. For instance, in order to accurately obtain the ionic and electronic conductivities of Li_1.3_Fe_1.2_Cl_4_, at different lithiation levels, Fu et al. simultaneously performed the EIS results to equivalent circuit fitting and Hebb‐Wagner methods, providing insights regarding the understanding of the reaction kinetics during the cycling process.^[^ ^21^ ^]^ Overall, understanding and regulating the evolution of mixed conductivity is the key to enabling stable, high‐performance redox‐active catholytes that can reliably support both ion and electron transport over extended cycling.

### 4.3 Dynamic Interface Evolution

The integrity of the interface between the cathode active material and the redox‐active catholytes is a critical determinant of battery performance, as it governs the interparticle charge‑transfer kinetics, suppresses parasitic side reactions, and absorbs the mechanical stress that further accumulates during cycling. Unlike traditional halide catholytes that act solely as Li‐ion conductors, redox‐active halide catholytes participate directly in redox reactions, imposing much stricter requirements on interfacial stability and compatibility. Repeated lithiation/delithiation processes induce cyclic volume changes in both cathode active material and catholytes, which can compromise intimate physical contact, and lead to increased interfacial resistance. Even worse, microcracks or voids form at the cathode active materials|catholyte interface due to repeated strain accumulation, which can exacerbate the contact loss. Although the inherent softness and deformability of halide catholytes can partially accommodate mechanical stresses, excessive deformation or creep still undermines structural integrity over extended cycles. To preserve interfacial contact, researchers pursue catholytes that exhibit minimal lattice breathing, or even self‐healing behavior. A representative example is LTG_0.25_PSSe_0.2_, whose near‐zero‐strain nature (≈1.2 % volume change) is too small to initiate cracking, thereby maintaining continuous contact even at high rates.^[^ ^60^ ^]^ Furthermore, Li_x_Fe_1.2_Cl_4_ exhibits gradually lower melting points and remarkable ductility upon charging, which enables a dynamic brittle‐to‐ductile transition that facilitates self‐healing during cycling (Figure 6d).^[^ ^21^ ^]^ Advanced characterization techniques such as focused ion beam–scanning electron microscopy (FIB‐SEM) cross‐sectional imaging, and operando synchrotron tomography provide further insights into interfacial evolution at micro‐ and nanoscale resolutions.^[^ ^105^ ^]^ Meanwhile, computational modeling of interfacial thermodynamics and mechanics can guide the design of more stable interfaces. Overall, achieving stable and dynamic interfacial contact is paramount for realizing the full energy and power potential of redox‐active halide catholytes.

## 5 Conclusion and Outlook

Redox‐active halide catholytes have emerged as a compelling route to unlock the higher energy density of ASSLBs. By combining fast Li‐ion transport with electronic conductivity that is 10^2^–10^5^ times higher than those of traditional halide catholytes, they alleviate electronic transport tortuosity and contribute to 10–50% additional reversible capacity at the cell level. Yet achieving simultaneously high mixed conductivity, redox reversibility, and cost efficiency remains challenging. Based on current understanding, we propose several perspectives to the potential research directions for these materials.

### 5.1 Discovery of New Materials

While light 3d metals such as Mn and Fe offer low mass and multiple oxidation states, the corresponding halides suffer from poor Li‐ion transport. Advanced synthesis routes that generate defect‐rich structures, including mechanochemistry, ultrafast quenching, and dopant‐driven disorder, should be combined with high‐throughput, data‐driven screening to identify composition‐defect chemistries capable of delivering Li‐ion conductivity above 1 mS cm^−1^.^[^ ^106^ ^]^

### 5.2 Dynamic Interface Evolution

A comprehensive understanding into phase transitions during cycling, along with the interfacial evolution between cathode active materials and redox‐active catholytes, is essential. Notably, certain phase transitions may facilitate a brittle‐to‐ductile transition, imparting self‐healing properties to the system.^[^ ^21^, ^107^, ^108^ ^]^ Furthermore, investigating cycling stability under low stacking pressures will be critical for advancing the practical application of ASSLBs.^[^ ^22^, ^109^ ^]^

### 5.3 Anionic Redox

Since the advent of Li‐ion batteries, transition metal cations have traditionally served as the primary redox centers. However, anionic redox activity, such as oxygen, has been demonstrated and widely accepted in layered oxide cathodes.^[^ ^110^, ^111^, ^112^ ^]^ Likewise, Chen et al. suggested that chloride ions can also undergo anionic redox reactions.^[^ ^31^ ^]^ The diverse valence states of chloride ions (from −1 to +7), coupled with their natural abundance, underscore the potential of anionic redox for redox‐active halide catholytes toward low‐cost, large‐scale energy storage technologies.

### 5.4 Anion‑Sublattice Engineering

While rigid anion frameworks offer foundational insights into classical ion transport mechanisms in halide SEs, harnessing anion dynamics opens transformative opportunities.^[^ ^88^, ^113^, ^114^ ^]^ Advanced computational and experimental evidence of the dynamic monkey bar and paddle‐wheel mechanisms point to a paradigm shift in the design principles of redox‐active halide catholytes. Future research should prioritize fine‐tuning of anion mobility, optimization of compositional and structural disorder, and integration of these advances with redox‐active functionalities to realize next‐generation ASSLBs with unprecedented performance.

### 5.5 Amorphous Halide Catholytes

Amorphous halide catholytes are emerging as a promising complement to crystalline counterparts, offering liquid‐like Li⁺/Na⁺ transport within flexible anionic framework and improved interfacial contact with active materials. Beyond conventional mechanochemical amorphization, composition‐guided strategies—particularly those incorporating redox‐active elements (as illustrated in Figure 4)—may enable the development of redox‐active amorphous halide catholytes. However, as catholytes participating in redox reactions, crystallization during (de)lithiation should be effectively suppressed, since it may lead to a significant decline in ionic conductivity.^[^ ^53^ ^]^

Collectively, these directions outline a roadmap for tuning redox‐active halide catholytes from conceptual promise to practical devices.

## Conflict of Interest

The authors declare no conflict of interest.
